# Centrosome-declustering drugs mediate a two-pronged attack on interphase and mitosis in supercentrosomal cancer cells

**DOI:** 10.1038/cddis.2014.505

**Published:** 2014-11-20

**Authors:** V Pannu, P C G Rida, B Celik, R C Turaga, A Ogden, G Cantuaria, J Gopalakrishnan, R Aneja

**Affiliations:** 1 Department of Biology, Georgia State University, Atlanta, GA, USA; 2 University of Gynecologic Oncology, Northside Hospital Cancer Institute, Atlanta, GA, USA; 3 Center for Molecular Medicine of the University of Cologne, Cologne, Germany

## Abstract

Classical anti-mitotic drugs have failed to translate their preclinical efficacy into clinical response in human trials. Their clinical failure has challenged the notion that tumor cells divide frequently at rates comparable to those of cancer cells *in vitro* and in xenograft models. Given the preponderance of interphase cells in clinical tumors, we asked whether targeting amplified centrosomes, which cancer cells carefully preserve in a tightly clustered conformation throughout interphase, presents a superior chemotherapeutic strategy that sabotages interphase-specific cellular activities, such as migration. Herein we have utilized supercentrosomal N1E-115 murine neuroblastoma cells as a test-bed to study interphase centrosome declustering induced by putative declustering agents, such as Reduced-9-bromonoscapine (RedBr-Nos), Griseofulvin and PJ-34. We found tight ‘supercentrosomal' clusters in the interphase and mitosis of ~80% of patients' tumor cells with excess centrosomes. RedBr-Nos was the strongest declustering agent with a declustering index of 0.36 and completely dispersed interphase centrosome clusters in N1E-115 cells. Interphase centrosome declustering caused inhibition of neurite formation, impairment of cell polarization and Golgi organization, disrupted cellular protrusions and focal adhesion contacts—factors that are crucial prerequisites for directional migration. Thus our data illustrate an interphase-specific potential anti-migratory role of centrosome-declustering agents in addition to their previously acknowledged ability to induce spindle multipolarity and mitotic catastrophe. Centrosome-declustering agents counter centrosome clustering to inhibit directional cell migration in interphase cells and set up multipolar mitotic catastrophe, suggesting that disbanding the nuclear–centrosome–Golgi axis is a potential anti-metastasis strategy.

Unlike *in vitro* cell cultures, cancer cells in patients' tumor tissues have low mitotic indices and proliferation rates.^1^ Consequently, drugs targeting mitosis demonstrate limited clinical efficacy, which exposes a fundamental weakness in the rationale underlying their clinical development. By contrast, classical microtubule-targeting agents (MTAs), largely believed to act by perturbing mitosis, remain the mainstay of chemotherapy in the clinic. Given the miniscule population of mitotic cells in patient tumors,^2, 3^ it stands to reason that MTAs must target interphase.^4^ This paradigm shift has spurred an intense search for novel interphase targets that combine the ‘ideal' attributes of cancer-cell selectivity and the ability to confer vulnerability on a large proportion of tumor cells.

Centrosomes, the major microtubule-organizing centers (MTOCs) of cells, are required for accurate cell division, cell motility and cilia formation.^5^ The number of centrosomes within a cell is strictly controlled, and their duplication occurs only once per cell cycle. Nearly all types of cancer cells have abnormal numbers of centrosomes,^6, 7, 8^ which correlates with chromosomal instability during tumorigenesis.^9, 10, 11^ Supernumerary centrosomes in cancer cells can cause spindle multipolarity and thus non-viable progeny. Cancer cells avoid this outcome by clustering centrosomes to assemble a pseudo-bipolar mitotic spindle, which yields viable daughter cells.^12^ Thus disrupting centrosome clustering may selectively drive cancer cells with amplified centrosomes to mitotic catastrophe and apoptosis without affecting normal cells.

The fate and interphase role of the supercentrosomal cluster inherited by each daughter cell at the end of a pseudobipolar mitosis is unknown. This is an important research question, because a majority of cells within tumors are in interphase and the centrosomes' command over microtubule nucleation is crucial for the cellular organization and motility in interphase. If cancer cells cluster centrosomes in interphase, then disrupting the cluster could impact interphase-specific processes, opening up a vital therapeutic avenue. We envision that centrosome declustering would (a) derail interphase-specific polarization and migration processes and (b) precipitate multipolar mitosis culminating in apoptosis. This two-pronged strategy would impact a significantly larger proportion of tumor cells and consign them to death. Our study herein establishes that centrosome-declustering drugs (RedBr-Nos, Griseofulvin and PJ-34) achieve this two-pronged attack as a unique class of agents that exhibit multiple cellular activities.

## Results

### High-grade cancers show robust centrosome amplification and clustering in interphase cells unlike cultured cell lines

**Figure 1:**
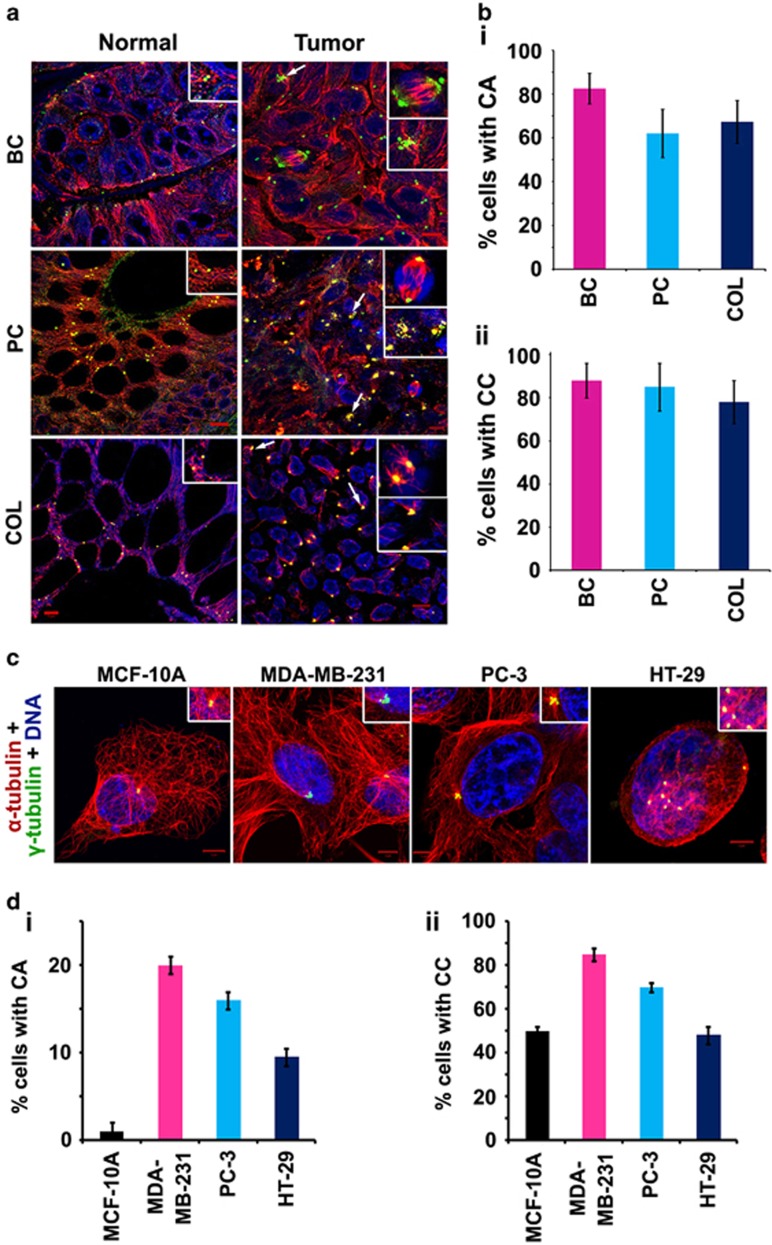
Clinical tumors show rampant centrosome amplification and clustering in interphase cells. (**a**) Representative immunofluorescence confocal micrographs showing centrosome amplification and clustering status in normal adjacent (left panel) and tumor tissues (right panel) from 10 patients of each cancer type. Insets show clustered centrosomes in representative mitotic cells (top inset) and interphase (bottom inset) in tumor samples and normal centrosomes in the normal samples. White arrows depict centrosome clusters in interphase cells. Centrosomes and microtubules were visualized by immunostaining for *γ*-tubulin (green) and *α*-tubulin (red), respectively. DNA was 4,6-diamidino-2-phenylindole (DAPI) stained (blue). (**b**i and **b**ii) Quantitative bar graphs representing the percentage of centrosome amplification and the percentage of interphase cells with amplified centrosomes that exhibit centrosome clustering, respectively, in the corresponding patient tissue samples. Centrosomes were counted in interphase cells from randomly selected fields totaling at least 200 cells per sample. (**c**) Representative immunofluorescence confocal micrographs showing centrosome amplification and clustering status during interphase in MCF-10A, MDA-MB-231, PC-3 and HT-29 cell lines. Insets show amplified and clustered/declustered centrosomes in interphase cells. Centrosomes and microtubules were visualized by immunostaining for *γ*-tubulin (green) and *α*-tubulin (red), respectively. DNA was DAPI stained (blue). (**d**i and **d**ii) Quantitative bar graphs representing the percentage of centrosome amplification and the percentage of cells with amplified centrosomes that exhibit centrosome clustering, respectively, in the corresponding cell lines. Centrosomes were counted in interphase cells from randomly selected fields totaling at least 200 cells per cell line. *P*<0.05. Scale bar, 5 *μ*m. BC=breast cancer, PC=prostate cancer, COL=colon cancer

We first assessed whether mitotic and interphase centrosome clusters are present in samples derived from high-grade carcinomas of the breast, prostate and colon. Contrary to the notion that high-grade cancers contain relatively large proportions of mitotic cells, we found that <2% of cells harbored mitotic spindles in the tumor samples examined (*n*=8 for each tissue). To assess centrosome amplification, we counted the number of *γ*-tubulin dots associated with 500 nuclei in each tumor sample. In most cases, centrosomes in tumor areas appeared significantly larger than centrosomes in adjacent uninvolved tissue. Exact centrosome numbers in these enlarged centrosomal clusters were difficult to determine owing to tight centrosome clustering. We therefore determined centrosomal volumes by measuring the *γ*-tubulin spots using the 3-D volume rendering function in the Zeiss imaging software (Axiovision LE). If the volume of a centrosome was determined to be >0.76 cubic micron (maximum volume of a centrosome observed in adjacent uninvolved tissue), it was considered a case of centrosome amplification. All tissue specimens showed centrosome amplification in 60–85% of tumor cells (Figures 1a and bi). In order to assess centrosome clustering in interphase, we counted centrosomes and measured centrosomal volumes in 500 interphase nuclei, and nuclei with >2 *γ*-tubulin spots or at least one *γ*-tubulin spot with increased volume at each MTOC were considered to show centrosome clustering. More than 75% of interphase cells exhibited centrosome clustering in all the cancer types examined (Figures 1a and bii).

In contrast, 6–18% of cancer cells in culture showed mitotic spindles (data not shown), which was significantly higher than the corresponding percentage in human tumors. We found that only 5–20% of cells in cultured cell lines exhibited amplified centrosomes (Figures 1c, di and dii), a lower frequency than that observed in patient tumors (Figure 1bi). We also observed that the multiple centrosomes in cell lines occurred as a juxtanuclear cluster in interphase cells (Figure 1c). Thus, while cancer cells in culture exhibit much higher levels of mitotic activity and lower levels of centrosome amplification compared with cancer cells within patients' tumors, cancer cells in culture and in tumors display the common features of centrosome clustering in interphase as well as in mitosis.

### Murine neuroblastoma cells constitute a good model system to study centrosome declustering

**Figure 2:**
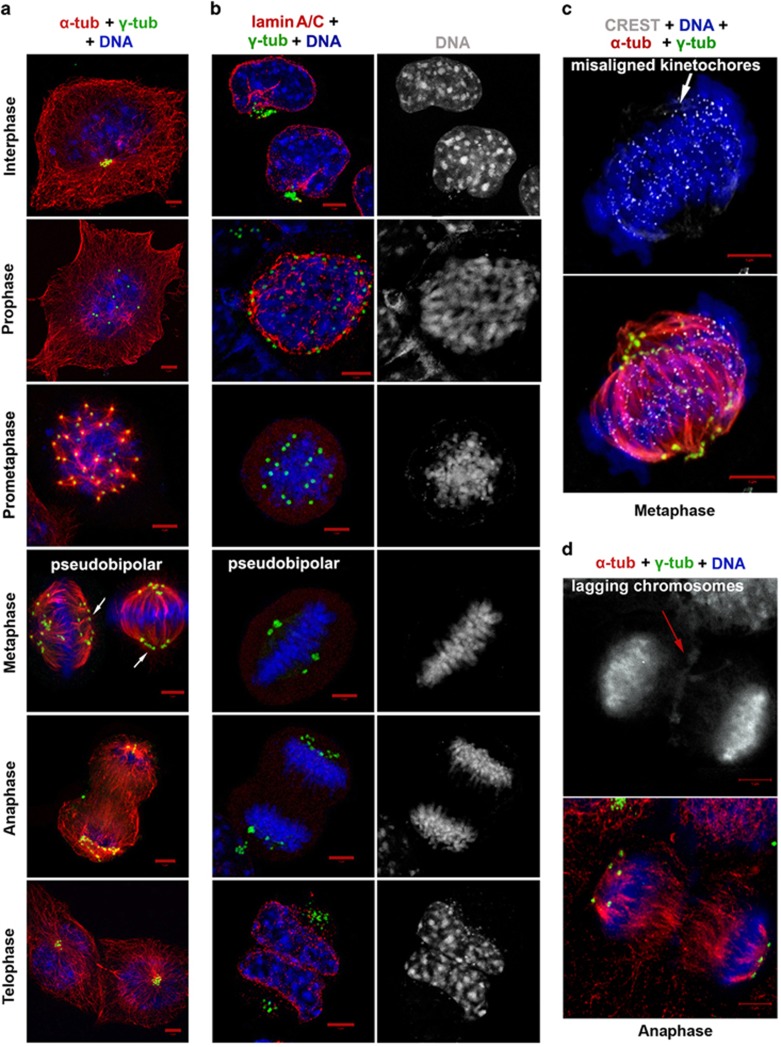
Cell cycle phase characterization of N1E-115 cells. (**a**) Representative immunofluorescence confocal micrographs depicting centrosome status in all cell cycle phases of N1E-115 cells. N1E-115 cells in interphase possess an enormous number of centrosomes as evident by *γ*-tubulin immunostaining (green). We acquired images as *z*-stacks with the slice interval of 0.40 *μ*m. *Z*-stack slices encompassing the entire depth of the cell were then merged, and *γ*-tubulin-positive spots were counted in interphase cells from randomly selected fields totaling 200 interphase cells. (**b**) Representative immunofluorescence confocal micrographs showing lamin A/C staining (red) across all cell cycle phases to visualize nuclear membrane in order to distinguish interphase declustering from prophase scattering of centrosome cluster. (**c**) Confocal micrograph of a metaphase cell stained with CREST antibodies (white), antibodies against *α*-tubulin (red) and *γ*-tubulin (green) and 4,6-diamidino-2-phenylindole (DAPI; blue) to detect microtubule-kinetochore attachments and DNA in a pseudobipolar mitotic spindle. (**d**) Representative micrograph of an anaphase cell immunostained for *α*-tubulin (red), *γ*-tubulin (green) and DAPI (blue) showing lagging chromosomes. Scale bar, 5 *μ*m

To identify an ideal *in vitro* model system to study interphase-specific centrosome-declustering events, we evaluated murine neuroblastoma N1E-115 cells. We found that 100% of N1E-115 cells harbor amplified centrosomes (5–20 centrosomes per cell). We also found that the centrosomal cluster in N1E-115 cells is a melange of single, free-standing mother and daughter centrioles and a few canonical centrosomes (Supplementary Figure S1). We thus wondered how these cells haul their centrosomal load through the cell cycle phases to accomplish cell division. In N1E-115 interphase cells, the multiple centrosomes localized as a distinct juxtanuclear cluster (Figures 2a and b). However, in ~10% cells, the multiple centrosomes showed significant scattering, and this feature correlated with chromatin condensation and the absence of a mitotic spindle. Lamin A/C immunostaining showed that cells with loose centrosome clusters still had an intact nuclear membrane. Thus these cells were confirmed to be in prophase (Figures 2a and b). About 30% of mitotic cells were in prometaphase (i.e., they lacked a nuclear membrane) and possessed multipolar spindles with multiple MTOCs at each spindle pole. Metaphase cells, by contrast, clustered supernumerary centrosomes into two polar groups to generate a pseudo-bipolar spindle (Figures 2a and b). The centrosomes at the poles of the spindle were often arranged in ‘ring-like' or ‘V-shaped linear' configurations (Figures 2a). Only ~20% cells displayed equal centrosome counts at the two spindle poles. CREST antibody labeling revealed that, in metaphase cells, the kinetochores did not line up immaculately along the spindle equator perhaps due to widespread merotelic attachments (Figure 2c). Centrosomes remained clustered at the two spindle poles through anaphase, and the occurrence of occasional lagging chromosomes indicated chromosome missegregation (Figure 2d). Telophase was marked by nuclear envelope reassembly around the decondensing chromatin and the inheritance of a juxtanuclear centrosomal cluster by each daughter cell (Figures 2a and b). These observations indicated that murine N1E-115 cells are a great model system to study centrosome clustering and declustering as they show both interphase and mitotic centrosome clustering similar to patient tumor cells.

In order to probe the mechanisms facilitating interphase clustering in N1E-115 cells, we examined the involvement of two major microtubule motors (human spleen embryonic tissue and testis (HSET) and dynein) in centrosome clustering in interphasic and mitotic N1E-115 cells. We found that siRNA knockdown of the kinesin-14 protein KifC1/HSET resulted in robust mitotic declustering generating ~65% multipolar mitotic cells, but negligible interphase declustering (15% as compared with 12% in vehicle-treated controls; Supplementary Figure S2). However, dynein inhibition by ciliobrevin treatment demonstrated substantial scattering of interphase centrosomal clusters (~50%) and considerable mitotic declustering (~35%) (Supplementary Figure S2). Based on these data, it seems that, while HSET is crucially involved in mitotic centrosome clustering, dynein has the major role in maintaining the centrosomal cluster during the subsequent interphase.

### Centrosome-declustering agents disperse interphase clusters and set the stage for a catastrophic mitosis

**Figure 3:**
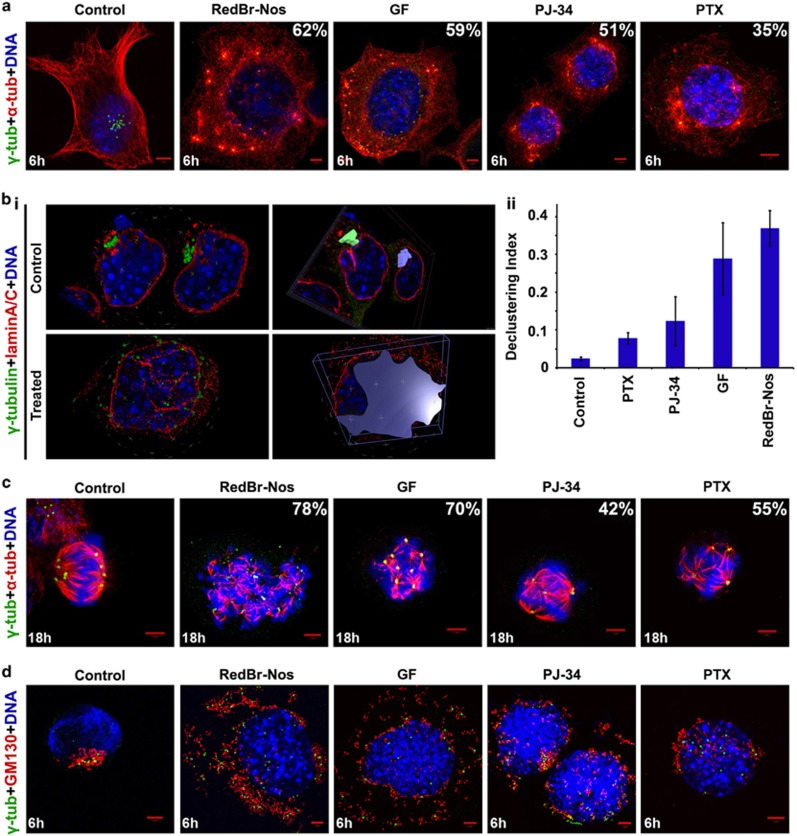
Interphase declustering induced by centrosome-declustering agents. (**a**) Confocal micrographs showing interphase declustering induced by 6-h treatment with RedBr-Nos (10 *μ*M), Griseofulvin (50 *μ*M), PJ-34 (25 *μ*M) and Paclitaxel (0.1 *μ*M). The percentages indicate proportion of interphase cells with declustered centrosomes. (**b**i) 3-D representation and quantitative volume analysis of control and drug-treated interphase cells using the Volocity 6.3 software. Cells were co-immunostained for lamin A/C (red) and *γ*-tubulin (green), and *z*-stacks were acquired with a 0.35 *μ*m *z*-step. *Z*-stack slices were then used to construct a 3-D image, and ROIs were defined to generate DI measurements. (**b**ii) Quantitative bar graph representing the DI of the four drugs. *P*<0.05. (**c**) Confocal micrographs showing spindle multipolarity induced by 18h treatment with declustering agents RedBr-Nos (10 *μ*M), Griseofulvin (50 *μ*M), PJ-34 (25 *μ*M) and Paclitaxel (0.1 *μ*M). Cells were co-immunostained for *α*-tubulin (red) and *γ*-tubulin (green). The percentages indicate proportion of mitotic cells with declustered centrosomes. (**d**) Confocal micrographs showing Golgi dispersal concomitant with interphase declustering upon 6-h treatment with all the four declustering agents at the stated concentrations. Cells were co-immunostained for GM130 (red) and *γ*-tubulin (green). DNA was DAPI (4,6-diamidino-2-phenylindole) stained. Scale bar, 5 *μ*m

Given the limited mitotic populations in human cancers, centrosome declustering during mitosis alone would fail to achieve sufficient elimination of cancer cells. On the other hand, interphase declustering may not only prime the cell for catastrophic mitosis but also ensure disruption of interphase-specific cellular processes that undergird migration. Thus we investigated how declustering agents affect centrosome clustering during interphase. We tested three declustering drugs (RedBr-Nos, Griseofulvin and PJ-34)^13, 14, 15, 16, 17^ and compared them with Paclitaxel, a tubulin-polymerizing drug. RedBr-Nos, Griseofulvin and Paclitaxel are known to bind tubulin^18, 19, 20^ but PJ-34 is a poly-ADP-ribose polymerase inhibitor with no known tubulin-binding property. However, they share common phenotypes, such as mitotic arrest and multipolar mitoses.^13, 14, 15, 16, 17, 21^ We found N1E-115 cells to be more sensitive to these drugs compared with other cancer cell lines (for instance, MDA-MB-231, HeLa) with IC_50_ values ranging between 0.05 *μ*M for Paclitaxel and 25 *μ*M for Griseofulvin (data not shown). To evaluate their effect on interphase clustering, we treated N1E-115 cells with drugs at their respective IC_50_ concentrations for 0, 3, 6 and 9 h and co-immunostained for *γ*-tubulin and *α*-tubulin to evaluate centrosomal spread and microtubule nucleation status, respectively (Figure 3a). RedBr-Nos and Griseofulvin inflicted more severe interphase declustering compared with PJ-34 and Paclitaxel. We also verified the cell cycle phases via lamin A/C immunostaining to distinguish interphase declustering events from prophase centrosomal spread. To quantitate the spread of the interphase centrosomal cluster, we generated a 3-D reconstruction of *z*-stack images of 25 randomly-selected interphase cells from the 6-h treatment group of each drug. By defining an ROI (region of interest) around the interphase centrosomal cluster, we calculated volume of the cluster spread using the Volocity software as shown in Figure 3bi. Likewise, defining an ROI using the cell periphery provided the cell volume. We defined the interphase declustering index (DI) for each drug as the ratio of the average volume of clusters to the average volume of the corresponding cell. Quantitative evaluation of DI revealed RedBr-Nos as the strongest declustering agent (DI=0.36), followed by Griseofulvin (DI=0.28) and PJ-34 (0.14). Paclitaxel showed the least declustering effect with a DI of 0.08 as compared with 0.02 in control cells (Figure 3bii). We also found that dispersal of the interphase centrosome cluster precipitated multipolar mitoses in the treated cells (Figure 3c). Again, the proportion of multipolar cells was higher in RedBr-Nos- and Griseofulvin-treated cells as compared with cells treated with PJ-34 and Paclitaxel, which mirrored the trend in interphase declustering (Figure 3c). These observations suggest that interphase declustering of centrosomes compels cells into catastrophic multipolar mitoses.

### Centrosome declustering in interphase disrupts Golgi coalescence and inhibits migration

The Golgi, which is primarily responsible for posttranslational modification and protein sorting, also functions as an MTOC.^22^ It has been hypothesized that supernumerary centrosomes may better organize the Golgi to enhance directional cell migration.^23^ We therefore investigated what happens to the Golgi upon declustering drug-induced dispersal of the interphase centrosomal cluster. We co-immunostained drug-treated cells for GM130 (a cis-Golgi matrix protein crucial for maintaining its structure) and *γ*-tubulin. Following treatment with declustering drugs, the interphase Golgi complex fragmented, and the distribution of Golgi fragments closely mimicked scattering of the centrosomal cluster, with the most robust effect seen with RedBr-Nos and Griseofulvin (Figure 3d).

**Figure 4:**
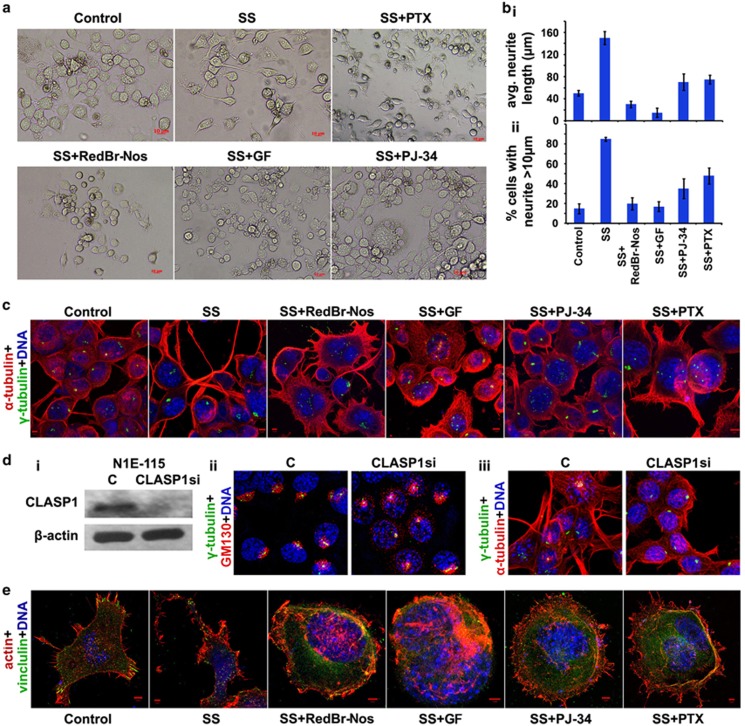
Inhibition of neuritogenesis by centrosome-declustering agents. (**a**) Phase-contrast images of N1E-115 cells in SS medium showing neurite formation after 48 h of SS or with RedBr-Nos (5 *μ*M), Griseofulvin (10 *μ*M), PJ-34 (10 *μ*M) and Paclitaxel (0.05 *μ*M) treatment. Scale bar, 10 *μ*m. (**b**i and **b**ii) Quantitative bar graphs representing the average length of neurites and the percentage of population of cells showing neurite length >10 *μ*m, respectively. Hundred cells were counted in each case. *P*<0.05. (**c**) Confocal micrographs showing neurite outgrowth after 48 h without SS, with SS or SS along with drug treatment, respectively. Cells were co-immunostained for *α*-tubulin (red) and *γ*-tubulin (green). DNA was 4,6-diamidino-2-phenylindole (DAPI) stained. (**di**) Immunoblot showing the CLASP1 expression levels in control and CLASP1 siRNA-transfected N1E-115 cells. (**d**ii) Confocal micrographs showing Golgi network immunostained for GM130 (red) and centrosome cluster immunostained for *γ*-tubulin (green) in control and CLASP1 siRNA-transfected N1E-115 cells. (**d**iii) Confocal micrographs showing neurite outgrowth in control and CLASP1 siRNA-transfected N1E-115 cells. (**e**) Confocal micrographs showing vinculin localization during neurite outgrowth after 48 h without SS, with SS or SS along with drug treatment, respectively. Cells were stained for F-actin using rhodamine-phalloidin, immunostained for vinculin (green) and DNA was DAPI stained. Scale bar, 5 *μ*m

Research suggests that Golgi-derived microtubules are not sufficient to preserve cell polarization; instead, they need to act in concert with the centrosome to establish and maintain cell polarization.^24^ In cancer cells harboring a supercentrosomal cluster, we predict that disrupting the cytoskeletal and organellar framework organized by a strongly polarizing supercentrosomal cluster will present a setback to the mechanical thrust that such a cluster can empower a migrating cell with; this in turn, we predict, will lead to impaired directional migration. As a surrogate for the polarization that underlies directional migration, we decided to examine neuritogenesis, a process in nerve cells involving the extension of polarized, elongated neurites. N1E-115 cells usually extend only one major neurite per cell, which can vary in length from 5 to 500 *μ*m. The growth cones of the neurites serve as primary focal points of motility. We evaluated the effect of declustering agents on cell motility by assessing the length and frequency of neurites formed in a serum-free medium on a laminin-coated surface. Neurite growth under these conditions is linear for up to 24 h, reaching a maximum around 36–48 h after plating. Phase-contrast imaging showed the presence of several elongated (10–200 *μ*m long) neurites upon 48 h of serum starvation (Figure 4bi). We observed 70–80% inhibition of neurite extension when treated with RedBr-Nos and Griseofulvin and moderate inhibition with PJ-34 and Paclitaxel treatment (Figures 4a and bii). Confocal imaging confirmed that inhibition of neurite formation was accompanied by dispersal of the interphase centrosome cluster, which is normally situated near the base of the tubulin-rich neurite shaft (Figure 4c).

In order to establish whether Golgi-dependent vesicular trafficking lies downstream of interphase centrosome clustering during cell polarization and neuritogenesis in N1E-115 cells, we studied the effect of centrosome-declustering-independent Golgi scattering on neuritogenesis. To accomplish this, we used CLASP1 siRNA to disrupt the Golgi-nucleated microtubules (Figure 4di), thus disarraying the directionality of post-Golgi vesicular trafficking but leaving the centrosome cluster intact, and evaluated whether these cells can generate neurites. We observed ~50% Golgi scattering upon CLASP1 knockdown (Figure 4dii). We observed that cells with CLASP1 siRNA formed significantly fewer neurites compared with control cells (Figure 4diii,Supplementary Figure S3C). This observation suggests that (i) disruption of Golgi network impedes Golgi polarization-dependent neuritogenesis, and (ii) Golgi complex integrity and polarized post-Golgi trafficking lie downstream of interphase centrosome clustering.

The spatio-temporal arrangement of Golgi apparatus serves as a geometrical regulator of cell migration as well as neurite extension. Thus we wanted to determine whether Golgi disruption upon CLASP1 knockdown affects cell shape and cell adhesion, modulation of which are crucial for cell migration as a precursory step for neurite extension in N1E-115 cells. We observed significant shift in the morphology of cells from majorly mesenchymal-like cell shape in cells transfected with control vector to largely amoeboid-like and more ‘rounded' cell shape in CLASP1 knockdown cells. This shift in cell morphology indicates changes in cell-substrate adhesion properties as a result of Golgi dispersal, which was confirmed by the reduction in vinculin localization at distinct adhesion focal points in CLASP1 siRNA cells (Supplementary Figure S3).

Vinculin stabilizes cell-substrate contacts in neuronal cells undergoing neuritogenesis,^25^ and activation by actin-binding proteins mobilizes vinculin to focal adhesions.^26, 27^ We therefore determined the localization of vinculin in the neurite extensions and the effect of declustering agents on its localization. To this end, we immunostained cells for vinculin and stained F-actin using rhodamine-phalloidin. Cells in serum-supplemented medium showed vinculin localization at focal adhesions with very little internalized vinculin. Upon serum starvation for 48 h, most of the vinculin was localized to the neurite growth cones. However, upon treatment of serum-starved (SS) cells with RedBr-Nos and Griseofulvin, we observed complete internalization of vinculin and complete loss of focal adhesion points. The observed effect was less severe with PJ-34 and Paclitaxel (Figure 4e). Centrosome-declustering drugs thus impair cell polarization and neurite formation and the localization of vinculin, a key player in the establishment of cell-substrate contacts. In order to support our rationale that the dispersion of centrosomal clusters in interphase is directly responsible for anti-migratory effects of these drugs and are not merely side effects of the drugs, we showed that the declustering agents were comparatively less affective in disrupting neuritogenesis of mouse neuroblastoma cells, Neuro-2a (harboring much lesser degree of centrosome amplification) (data shown in Figure S4).

These observations underscore the immense clinical potential of centrosome declustering as a selective therapy for cancer cells harboring excess centrosomes, without affecting cells with normal centrosome content.

### Inhibition of migration results in interphase cell death or pushes cells into catastrophic mitosis

**Figure 5:**
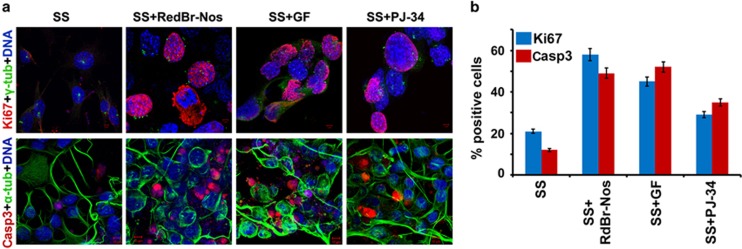
Inhibited migration induces interphase cell death or pushes cells into catastrophic mitosis. (**a**) Confocal micrographs showing proliferative cells with 24 h of SS or SS along with RedBr-Nos (5 *μ*M), Griseofulvin (10 *μ*M) and PJ-34 (10 *μ*M) treatment, respectively. Cells were co-immunostained for Ki67 (red) and *γ*-tubulin (green). The percentages show proportion of Ki67-positive cells. Scale bar, 5 *μ*m. Confocal micrographs showing cells undergoing apoptosis with 9 h of SS or SS along with RedBr-Nos (5 *μ*M), Griseofulvin (10 *μ*M) and PJ-34 (10 *μ*M) treatment, respectively. Cells were co-immunostained for cleaved caspase-3 (red) and *α*-tubulin (green). E percentages show proportion of cells that stained positive for cleaved caspase-3. Scale bar, 10 *μ*m. (**b**) Quantitative bar graphs representing the percentage of Ki67- and caspase-3-positive cells when treated with the respective drugs. Two hundred cells were counted in each case. *P*<0.05

Several studies suggest an intrinsic, inverse relationship between cell migration and cell proliferation. This concept that cells exist in mutually exclusive cellular states that either permit motility or mitotic activity is evidenced by numerous *in vitro* and *in vivo* studies^28^ and is referred to as ‘Go-or-Grow'.^29^ We thus explored whether inhibiting migration via declustering drug treatment of SS N1E-115 cells enhances proliferation (indicated by Ki67 nuclear immunostaining) or induces apoptosis (indicated by cleaved caspase-3 immunostaining). We found a high proportion of Ki67-positive cells upon treatment with the three declustering drugs (RedBr-Nos, Griseofulvin and PJ-34) when compared with negligible number of Ki67-positive SS N1E-115 cells, which should predominantly be in the G0 phase of the cell cycle (Figure 5, top panel). These data suggest that declustering drugs cause more cells to enter the cell cycle under the conditions of serum starvation. We also wanted to explore whether apoptosis is induced by interphase declustering and whether any induced cell death depends on the cells' passage through mitosis. Cleaved caspase-3 staining in N1E-115 cells upon treatment with the three drugs for 9 h (a time point at which the vast majority of cells were in interphase; data not shown) revealed a higher proportion of caspase-3 positive interphase cells in the drug-treated cultures compared with untreated controls, indicating significant induction of cell death during interphase (Figure 5, bottom panel). Interphase-specific cell death was confirmed with a cell-clock assay (as described in the Supplementary Data and Supplementary Figure S5). These observations suggest that disrupting the supercentrosomal cluster during interphase in N1E-115 cells (a) induces interphase catastrophe, and (b) pushes cells into a proliferative mode leading to a catastrophic mitosis. These data thus support the notion that centrosome-declustering drugs launch a two-pronged attack on supercentrosomal cells.

## Discussion

Majority of cancer patients succumb to cancer due to metastases for which effective therapeutic options are currently lacking. Cell migration and invasion are the key cell biological processes that underlie metastatic dissemination of cancer cells. With the recent realization that patient tumors are slow growing with doubling times ranging between 100 and 700 days, the glory of mitosis as a target has faded.^1^ Most anti-mitotic drugs have failed so far in clinical trials either owing to limited efficacy, as most cells in patients' tumors are not mitotic, or excessive toxicity; thus interphase is a more promising chemotherapeutic target.

Recent studies have provided *in vitro* evidence that centrosome amplification can cause oncogene-like effects in promoting cellular invasion in mammary epithelial cells. These findings assert that structural alteration of the cytoskeleton via centrosome amplification confers transformation potential to normal epithelial cells and is directly responsible for tumor initiation and progression.^30^ Our study herein is the first to demonstrate that interphase cancer cells in patients' tissues organize their excessive centrosomal load in the form of a juxtanuclear supercentrosomal cluster. This tight cluster is maintained throughout interphase and disperses only transiently during prophase followed by reclustering. We show that untimely dispersal of the supercentrosomal cluster in interphase drastically impacts cytoskeletal and organellar organization; in particular, the Golgi fragments and each dispersed centrosome carries with it a group of associated Golgi fragments. As a consequence, the cells are no longer able to produce neurite extensions and establish proper focal contacts with the substrate, as needed for directional migration. Interphase clustering of supernumerary centrosomes is thus a cancer-specific trait that may help cancer cells survive and migrate. We assert that a powerful strategy to cripple the migratory agenda of cancer cells is to disrupt the centrosomal cluster by using centrosome-declustering agents.

The importance of interphase clustering to cancer cells is spotlighted by the dire consequences of disrupting the interphase centrosomal cluster. It has been established that polarity of the Golgi complex and directionality of Golgi-nucleated microtubule arrays are crucial for directional cell migration. The correct orientation and positioning of the Golgi apparatus is regulated by the interplay of various factors, including both centrosome- and Golgi-derived microtubules, and the binding of A-kinase anchor protein 450 (AKAP450) to GM130, gamma-tubulin ring complex and dynein–dynactin complex.^31^ Our data confirms the scattering of GM130 accompanied by centrosomal cluster scattering upon action of declustering agents, indicating possible scattering of AKAP450 as well. Thus disrupting the centrosomal cluster leads to concurrent scattering of the Golgi apparatus as a result of which Golgi-derived microtubules and post-Golgi vesicular trafficking are no longer focused toward the leading edge, which may have a dramatic effect on directional cell migration.

Thus centrosome-declustering drugs launch a two-pronged offensive on supercentrosomal cancer cells in that they not only scatter the centrosomes through the cytoplasm and profoundly disrupt the Golgi network to impede cell migration in interphase but also effectively trap cancer cells in a non-resolvable state that culminates in spindle multipolarity and metaphase catastrophe in mitosis. We are confident that the observed phenotypes are triggered by centrosome cluster dispersal and not due to the drugs used, as the declustering drugs in our study function through very different mechanisms and yet produce similar phenotypes.

Our study demonstrates that mouse neuroblastoma cells N1E-115 are an excellent test-bed for studying the mechanisms and effects of centrosome clustering and declustering. These cells, unlike other cell lines, have 100% centrosome clustering in interphase and ~90% ‘pseudobipolar' spindle formation in mitosis. A ‘good' declustering drug should be able to scatter its megacentrosomal cluster into an unrestricted pool of centrioles in this cell line, consequently generating excessive spindle multipolarity and severe, death-inducing aneuploidy in daughter cells. The DI as described for N1E-115 cells in our study can facilitate quantitative comparison of the efficacy of putative declustering agents. Based on our data, RedBr-Nos and Griseofulvin showed more dramatic effects on centrosome declustering and inhibition of neurite formation as compared with PJ-34 and Paclitaxel. In sum, our findings reveal the previously underappreciated aspects of the actions of centrosome-declustering drugs, their potential application as anti-metastatics and the importance of interphase as a chemotherapeutic target.

## Materials and Methods

### Cell culture and transfection

N1E-115 mouse neuroblastoma cells (CRL-2263) were purchased from the American Type Culture Collection (Manassas, VA, USA). Cells were cultured in Dulbecco's modified Eagle's medium (DMEM) with 4.5 g/l glucose, 1.5 g/l sodium bicarbonate and supplemented with 10% FBS. Cells were harvested by incubating them in Modified Puck's Saline D1 solution at room temperature until the cells detached. MCF-10A cells were cultured in MEGM medium (Lonza, Basel, Switzerland), MDA-MB-231 and HT-29 cells in DMEM and PC-3 cells in RPMI medium supplemented with 10% FBS. All lines were tested and were free of *Mycoplasma* contamination. N1E-115 cells were transfected with X-tremeGENE siRNA transfection reagent (Roche, Basel, Switzerland) according to the manufacturer's instructions.

### Cellular protein preparation, western blotting, immunofluorescence and antibodies and other reagents

Cells were cultured to ~70% confluence, and protein lysates were collected following drug treatment, transfection or otherwise for western blotting following methods described in previous publications.^13^ For immunofluorescence staining, cells grown on glass coverslips were fixed with cold (−20 °C) methanol or 4% paraformaldehyde (room temperature) for 10 min and blocked by incubating with 2% bovine serum albumin/PBS/0.05% Triton X-100 at 37 °C for 1 h. Specific primary antibodies were incubated with coverslips for 1 h at 37 °C at the recommended dilution followed by 1 : 2000 dilution of Alexa 488- or 555-conjugated secondary antibodies. Antibodies against *γ*-tubulin, *α*-tubulin and *β*-actin were from Sigma (St Louis, MO, USA); cleaved caspase-3 was from Cell Signaling (Danvers, MA, USA). Alexa 488- or 555-conjugated secondary antibodies were from Invitrogen (Carlsbad, CA, USA). Anti-Ki67 antibody was from Abcam (Cambridge, MA, USA). Anti-GM130 antibody was from BD Biosciences (San Jose, CA, USA). Anti-vinculin antibody was purchased from Millipore (Billerica, MA, USA). Anti-lamin A/C, anti-centrin-2 and anti-CLASP1 antibodies and horseradish peroxidase-conjugated secondary antibodies were from Santa Cruz Biotechnology (Santa Cruz, CA, USA). CLASP1 siRNA was ordered from Origene (Rockville, MD, USA). SMARTpool: ON-TARGETplus KIFC1 siRNA (Dharmacon, Pittsburgh, PA, USA) was used to knockdown HSET in N1E-115 cells.

### Electron microscopy

N1E-115 cells were grown on coverslips made of Aclar film (Electron Microscopy Sciences, Hatfield, PA, USA) and were processed for electron microscopy as described in previous publication.^32^

### Neurite extension assay

N1E-115 cells were grown on glass coverslips coated with laminin in 35-mm tissue culture dishes and treated with the respective drugs diluted in serum-free medium; the cells were then fixed, labeled and visualized by immunofluorescence microscopy. Cells bearing neurite-like structures with a length of at least one cell diameter were identified by immunofluorescence microscopy using an *α*-tubulin antibody. At least 200 cells were counted for each condition, and the experiments were repeated three times. The images were taken using a Ziess LSM 700 confocal microscope (Oberkochen, Germany).

### Cell-clock assay

N1E-115 cells were grown to 60–70% confluence and then treated with Griseofulvin, RedBr-Nos or PJ-34 for 3 and 6 h. After the end of treatment, the cell-clock dye (prewarmed at 37 °C) was added (150 *μ*l per well in 12-well plate), and the cells were incubated at 37 °C for 1 h. The dye was washed twice with prewarmed DMEM medium, and PI was added for 15 min at room temperature and washed twice with PBS. Fresh medium was added, and the cells were imaged in bright field (to assess the different phases of cell cycle) and fluorescent (red for PI) channel. Cell-clock dye is a redox dye, which is readily taken up by live cells. In G1 phase, the dye in its reduced form is yellow in color, while in the intermediate state it is green (S and G2 phases) before turning dark blue in the fully oxidized form (mitosis). Micrographics taken in the bright field channel depicts cells in different cell cycle phases based on their respective colors.

## Abbreviations

DI: declustering index
MTA: microtubule-targeting agent
MTOC: microtubule-organizing center
ROI: region of interest
SS: serum-starved
AKAP: A-kinase anchor protein
HSET: human spleen embryonic tissue and testis
