# Supplementary material for: Centrosome-declustering drugs mediate a two-pronged attack on interphase and mitosis in supercentrosomal cancer cells
Source: Cell Death Dis. 2014 Nov 20;5(11):e1538–. doi: 10.1038/cddis.2014.505 (PMC4260758; doi:10.1038/cddis.2014.505)
Supplement: Supplementary Information [file cddis2014505x1.doc]

**Supplementary Data**

**The centrosomal cluster in N1E-115 cells is a melange of single mother and daughter centrioles and a few canonical centrosomes**

We wanted to delineate if γ-tubulin spots observed in N1E-115 cells are “real” centrosomes containing centrioles or just γ-tubulin-containing foci since γ-tubulin is a major pericentriolar material (PCM) component. We therefore co-immunostained N1E-115 cells for (i) a specific centriolar protein, centrin-2, and (ii) cenexin/Odf2, a mother centriole appendage protein which accumulates on the new mother centriole during G2/M. Intriguingly, we found that majority of centrosomes were single, freestanding mother centrioles (centrin-2-positive, cenexin-positive), with a small proportion of centrin-2-positive single daughter centrioles **(Suppl. Fig.1A, Cell A, B, C)**. Quantitation of daughter and mother centrioles in 200 interphase cells from 15 randomly-selected fields showed ~85% mothers and ~10% daughters. Each cell also had 1-2 “canonical centrosomes” with both mother and daughter centrioles. Transmission electron microscopy (TEM) analysis of serial sections showed that the freestanding centrioles were all randomly oriented with respect to each other and were clustered near the cell nucleus **(Suppl. Fig. 1B)**. Some of these single centrioles bore distal appendages (red arrows) typical of mature mother centrioles **(Suppl. Fig. 1B)**. In addition, TEMs showed 1-2 “canonical” centrosomes in each cell with a typical orthogonal configuration between parental and daughter centrioles, and the mother centriole displaying distal appendages **(Suppl. Fig. 1B, white arrows)**. These data concur with earlier reports(1). Our data strengthen the idea that centrosome clustering does not necessarily require centrosomes to be “canonical”; instead, it appears that as long as the centrioles can nucleate microtubules and function as MTOCs, cellular mechanisms ensure their clustering through the major part of the cell cycle.


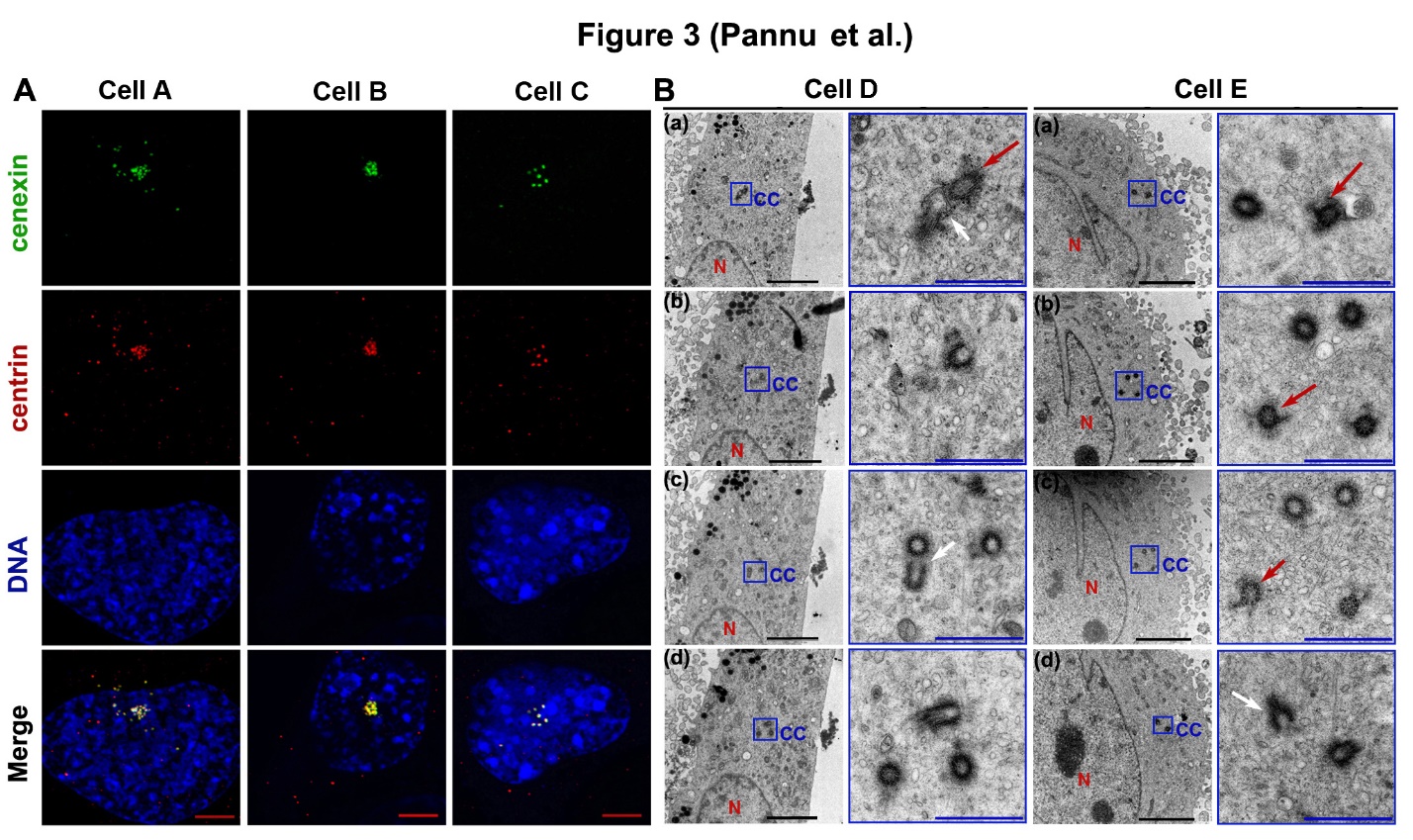


**Suppl. Fig. 1.**  **Composition of centrosomal clusters in interphase N1E-115 cells.** **A.** Immunofluorescence confocal micrographs showing red centrin-2 (daughter) and green cenexin (mother) dots in three representative N1E-115 cells in interphase (Cell A,B,C). DAPI-stained DNA is in blue. Scale bar, 5 µm. **B.** Thin-section electron micrographs of 2 representative cells (Cell D, E) are arranged in serial sequence from (a) to (d), showing single mother centrioles with appendages (red arrow), single daughter centrioles without appendages and canonical centrosomes (white arrow) near the nucleus (N). Scale bar, 1 µm. Low-magnification images in the left panels show the proximity of the centrosome cluster (CC) to the nucleus (N). Higher magnification images of the regions within the blue boxes are shown on the right-hand side panels in (a)-(d).


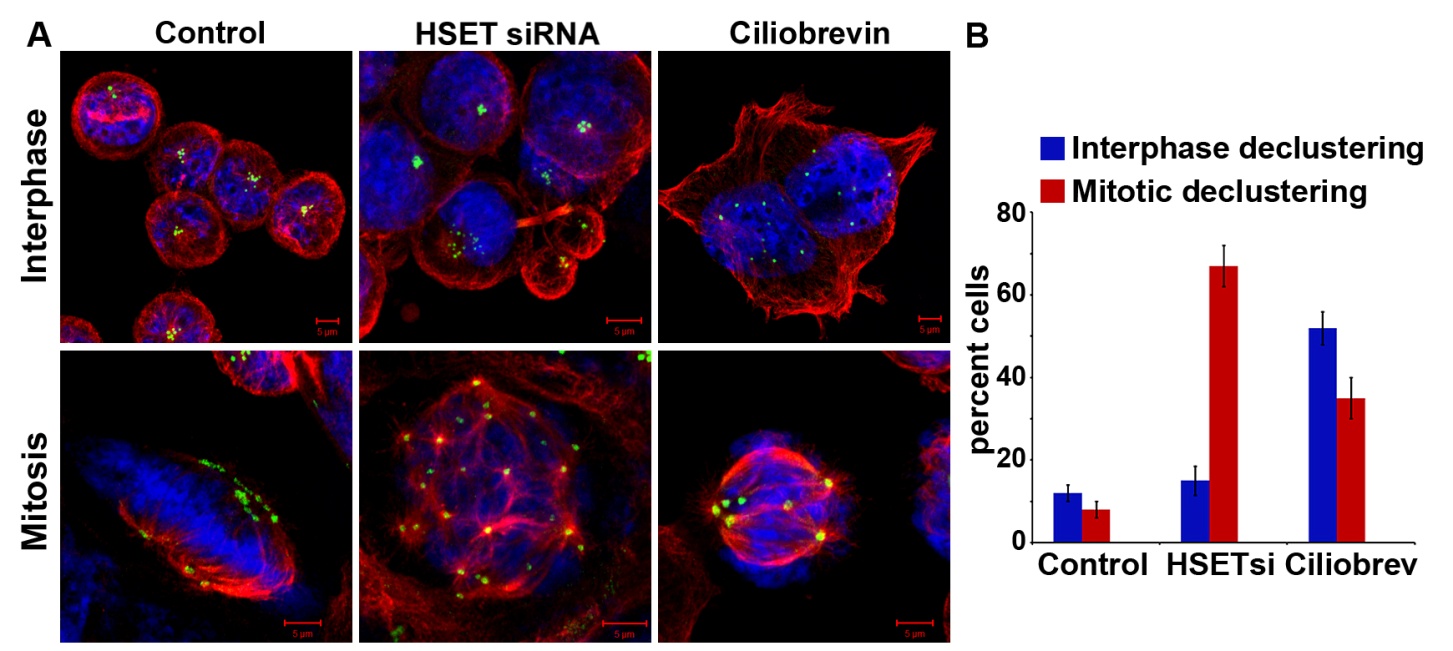


**Suppl. Fig. 2:** **Involvement of microtubule motors in interphase and mitotic declustering. A.** Confocal micrographs showing interphase (top panel) and mitotic (bottom panel) N1E-115 cells upon treatment with vehicle, HSET siRNA or Ciliobrevin (50µM for 16h). Cells were co-immunostained with α-tubulin (red) and γ-tubulin (green). DNA was stained with DAPI. Scale bar, 5µm. **B.** Quantitative bar graphs representing percentage of total cells that show interphase or mitotic declustering upon treatment with vehicle control, HSET siRNA or Ciliobrevin. p<0.05


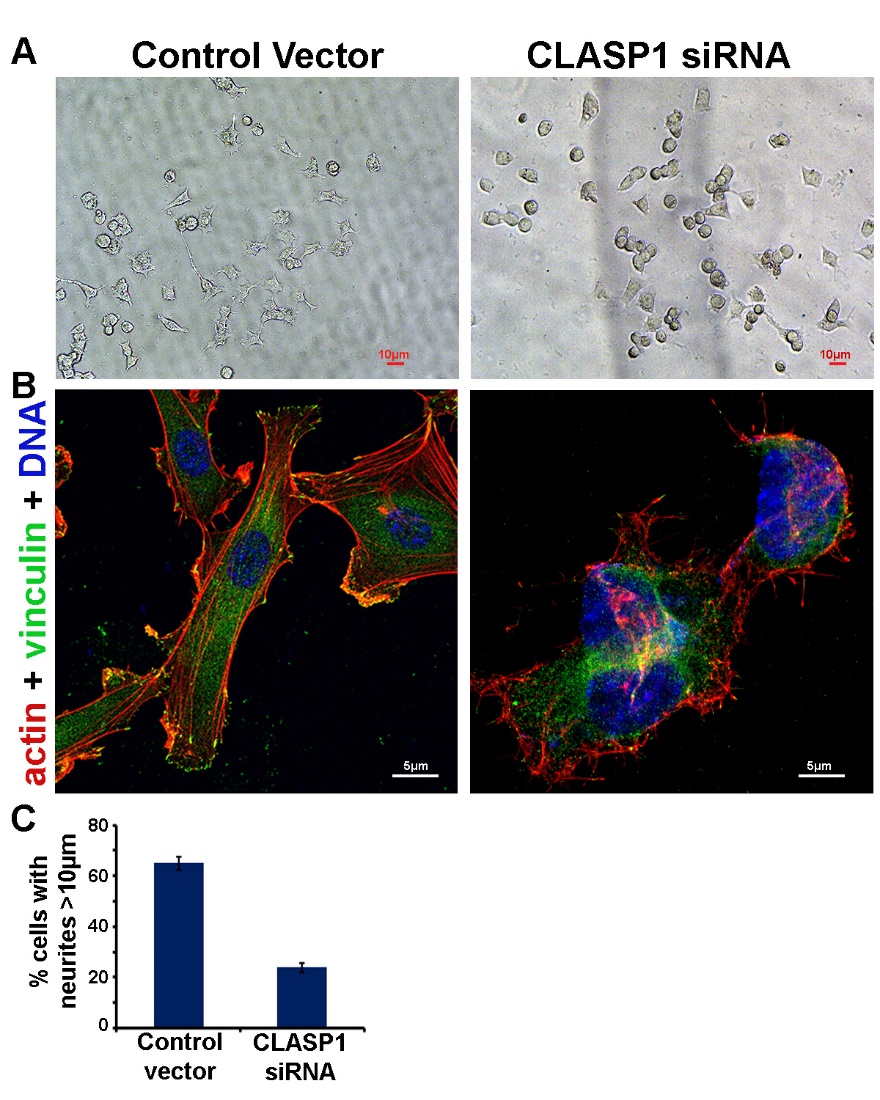


**Suppl. Fig. 3:** **Changes in cell-substrate adhesion and cell shape as a result of Golgi dispersal.** A. Phase contrast images of N1E-115 cells transfected with control vector (CV) or CLASP1 siRNA. Scale bar 10µm. **B.** Confocal micrographs showing vinculin localization upon transfection with CV or CLASP1 siRNA. Scale bar 5µm. **C.** Quantitative bar graphs representing percent population of cells showing neurite length >10 µm, respectively. 100 cells were counted in each case. p<0.05.

**Centrosome declustering in interphase inhibits cell migration**

In order to support our rationale that the dispersion of centrosomal clusters in interphase is directly responsible for anti-migratory effects of these drugs and are not merely side effects of the drugs, we examined if these declustering agents were able to affect neuritogenesis of cells with normal centrosomes. To this end, we used mouse neuroblastoma cells, Neuro-2a harboring a much lesser degree of centrosome amplification (limited to 10-15%) and assessed the effect of declustering drugs on the ability of these cells to grow neurites upon serum starvation, as described earlier (**Suppl. Fig. 4A,B**). This allowed us to determine the selectivity of these drugs exclusively against cells harboring extra centrosomes. We observed only ~20% inhibition of neurite extension when treated with RedBr-Nos and Griseofulvin and ~15% inhibition with PJ-34. As shown in **Suppl Fig. 4**, declustering agents affected neurite formation selectively in cells with extra centrosomes (denoted by white arrows). However, Paclitaxel treatment resulted in ~80% inhibition of neurite extension, which is considerably higher than the declustering drugs suggesting that the mode of action of paclitaxel is independent of the centrosomal status. Due to significant perturbation of microtubule ultrastructure, tubulin polymerization by paclitaxel resulted in extensive neurite inhibition (**Suppl. Fig. 4A, B**). In Neuro-2a cells, treatment with declustering drugs did not cause the cell shape and vinculin localization changes that we saw in N1E-115 cells (data not shown), suggesting that Golgi integrity and directionality of post-Golgi vesicular trafficking was unaffected in these cells.


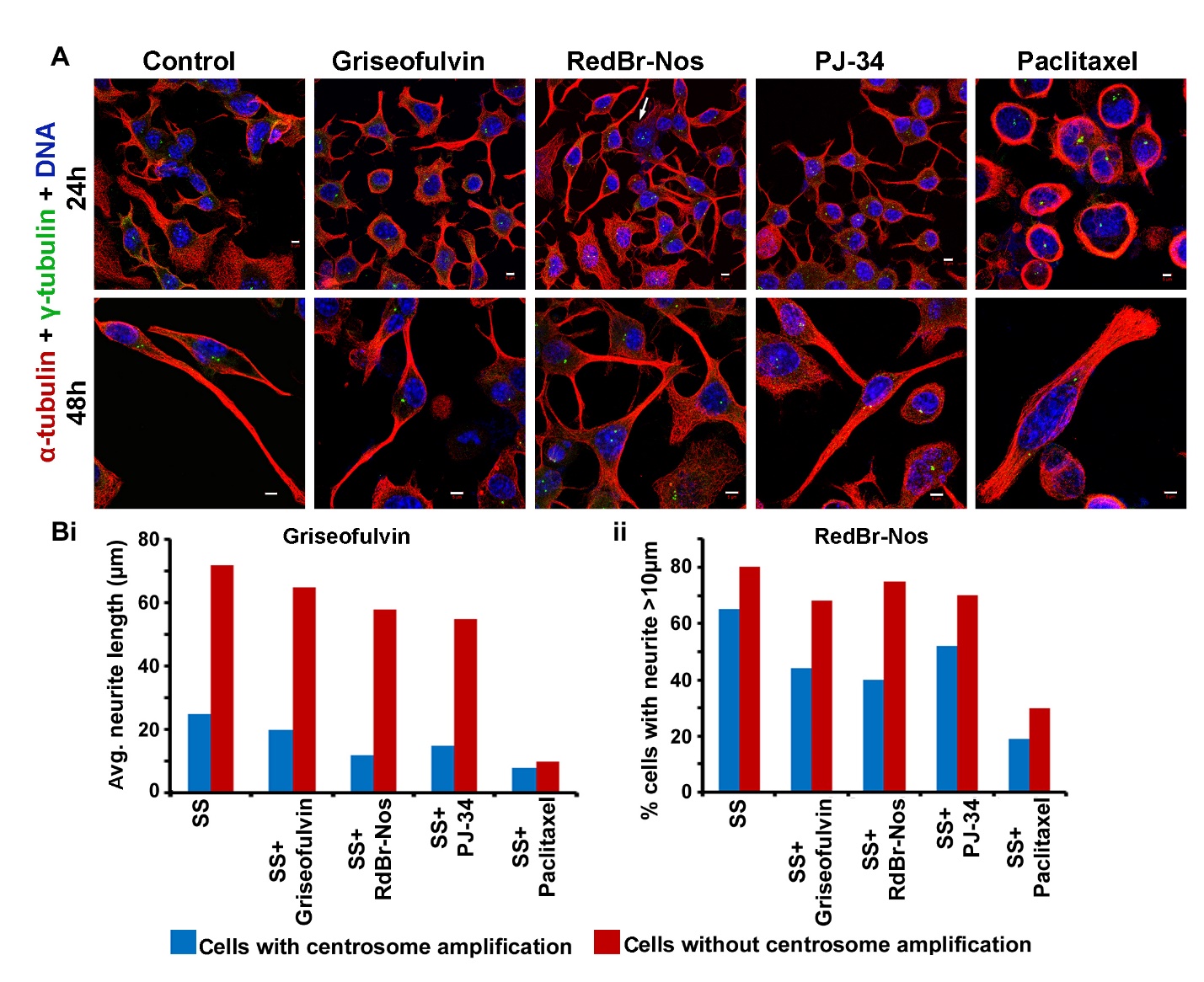


**Suppl. Fig. 4:** **Effect of centrosome declustering drug treatment on Neuro-2a cells. A.** Confocal micrographs showing neurite outgrowth in Neuro-2a cells after 48h with Serum starvation (SS), or SS along with drug treatment, respectively. Cells were co-immunostained for α-tubulin (red) and γ-tubulin (green). DNA was DAPI-stained. Scale bar 5µm. **Bi,ii.** Quantitative bar graphs representing average length of neurites and percentage of cells showing neurite length >10 µm, respectively. 100 cells were counted in each case. p<0.05.

**Inhibited migration results in interphase cell death**

To demonstrate the induction of cell death in interphase upon drug treatment, we performed cell-clock assay to identify the distinct cell cycle phases exhibiting cell death. To monitor cell death, we stained the cells with Propidium iodide (PI) (0.2ug/ml). PI is taken up by cells following the disruption in cell membrane integrity, thus indicating dead cells. Upon drug treatments for 3h and 6h, we observed G1, S and G2-phase cells undergoing cell death (**Suppl. Fig. 5**). Only a negligible proportion of M-phase cells were found PI positive at these time points (1-2%). The appearance of cell death as early as 3-6h of drug treatment in conjunction with the determination of cell cycle phase confirm the interphase-specific cell death upon treatment with declustering agents.


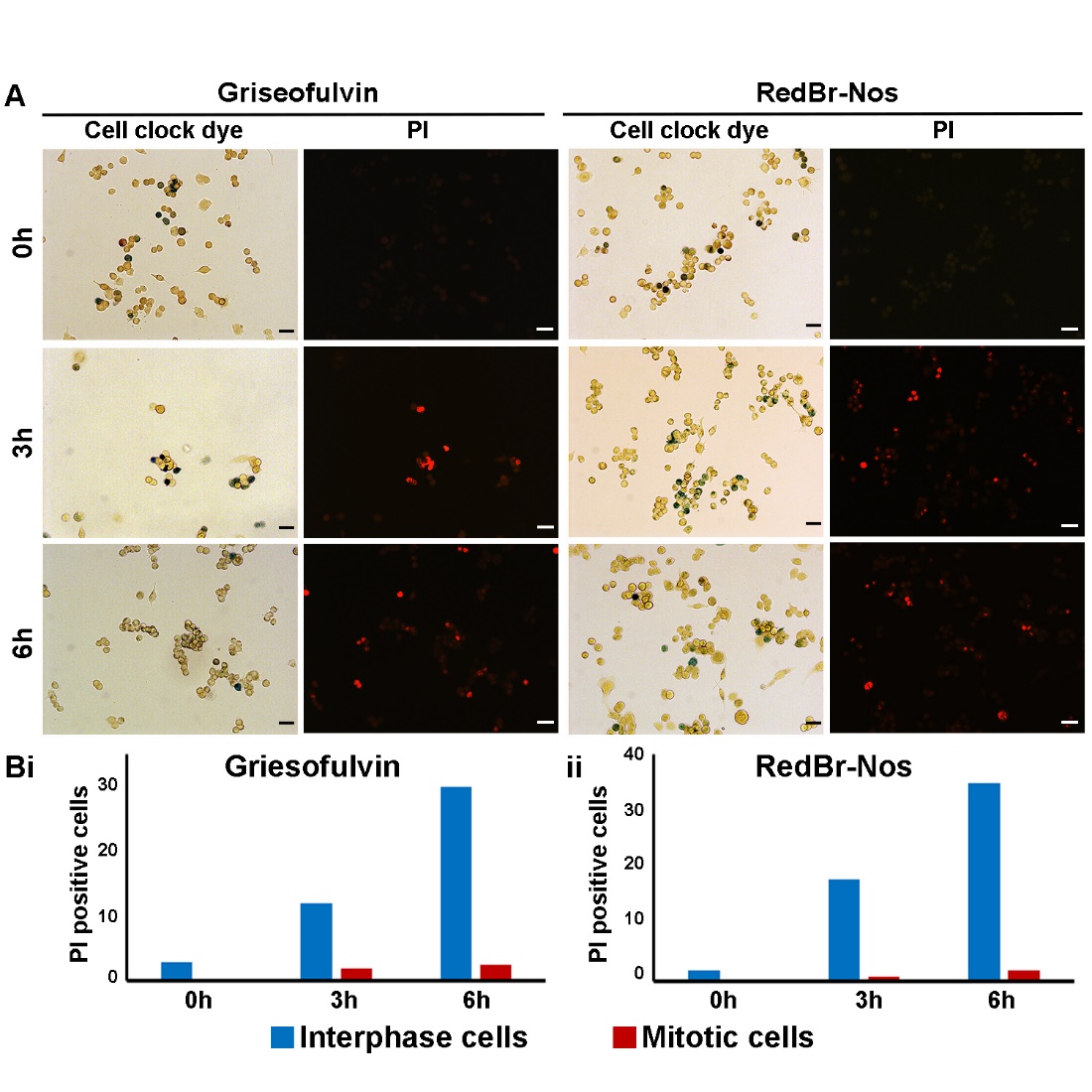


**Suppl. Fig. 5.** **Induction of cell death in interphase, but not in mitosis.** **A.** Micrographs showing bright field (left panel) and fluorescent (right panel) images of N1E-115 cells treated with Griseofulvin and RedBr-Nos, respectively. Bright field images depict different cell cycle phases represented by different colors of the Cell Clock dye. Fluorescent images depict the PI staining in the same field. Scale bar 10µm. **Bi,ii.** Quantitative bar graphs representing propidium iodide (PI) positive cells at various time points when treated with Griseofulvin and RedBr-Nos, respectively. 200 cells were counted in each case. p<0.05.

**REFERENCES**

1. Gisselsson D, Hakanson U, Stoller P, et al. When the genome plays dice: circumvention of the spindle assembly checkpoint and near-random chromosome segregation in multipolar cancer cell mitoses. PLoS One 2008; 3: e1871.
